# Local Anæsthesia

**Published:** 1901-05-18

**Authors:** 


### LOCAL ANÆSTHESIA.

With the improvements that have taken place in the technique of many abdominal operations, and with the lessening of the mortality due directly to such operations, the danger of the anæsthesia in certain cases has become a matter of growing importance. It is especially where shock is to be feared that this risk becomes a serious one, and it is in such cases that the question of employing local instead of general anæsthesia is one of considerable interest. So far as the mere prevention of pain is concerned, the anæsthesia need not be very deep, and experience is accumulating in favour of the use of eucaine, or even simple skin freezing, as a means of producing the required numbing of the part to be incised. Thomas H. Morse,[1] of Cromer, records several cases of abdominal operation under local anæsthesia. In two cases of ruptured tubal pregnancy the skin was frozen with anestile, and the only painful part of the operation was the dragging on the broad ligament for the purpose of pulling the ovary and tube up into the wound in order to ligature and remove them. In one case the patient complained more of pain from the heat of the water (110° Fahr.) used for washing out the clots than from all the rest of the manipulations. In a case of strangulated femoral hernia, the skin was frozen with ethyl chloride, and the operation was accomplished with very little pain; there was no shock and recovery was uninterrupted. The subject of local anæsthesia is one which will well repay further investigation. That a very useful degree of anæsthesia can easily be produced is well enough known, but it is not yet quite certain how far it can be trusted; and that is the difficulty. Simple as it may seem to use local anæsthetics for long operations, when these are done in hospital theatres, with general anæsthesia always available in case there should be any hitch, it is rather different in the country. We have hardly yet arrived at quite such a stage of certainty regarding the extent and depth of local anæsthesia as to make us entirely happy in attacking deep structures under such circumstances that, were the local anæsthetic to fail, we should find ourselves thrown back into pre-anæsthetic days—no joke in the midst of a laparotomy.

[1] Lancet, May 11.

